# “With a husband like that, I’d be happy to have three kids”: ideal fatherhood, endogenous symbolic annihilation of motherhood, and fertility intentions on Chinese social media

**DOI:** 10.3389/fpsyg.2026.1847967

**Published:** 2026-08-10

**Authors:** Zheyou Ye, Huaming Chen

**Affiliations:** College of Journalism, Communication and Publishing, Sichuan University, Chengdu, China

**Keywords:** coparenting, fatherhood performance, fertility intention, social cognition, symbolic annihilation

## Abstract

**Background:**

Chinese social media has become an important arena where fatherhood is made visible as a form of coparenting. In the context of demographic transition, Confucian gender norms, and rising feminist consciousness, short videos of fathers caring for children often attract public praise. Yet within fatherhood-centered comment threads, such praise often positions maternal labor as an assumed background rather than an equally recognized form of care work. Existing research has examined platform content and fatherhood discourse, but less is known about how everyday user interaction reshapes the value hierarchy of paternal and maternal labor.

**Methods:**

This study analyzes 2,798 valid public comments collected from Xiaohongshu using the keyword “daddy raising kids.” It combines thematic analysis, critical discourse analysis (CDA), and symbolic annihilation theory to examine the implicit biases and social meanings embedded in these discursive practices.

**Results:**

The analysis identifies four main themes. First, users construct the “ideal father” by spectacularizing male caregiving and positioning maternal labor as an assumed background rather than an equally recognized form of care work. Second, fatherhood is given performative and commercial value, while motherhood is often positioned as less visible and more naturalized. Third, women’s “conscious resistance” coexists with “compliant discourse” that reaffirms traditional gender roles. Fourth, conditional fertility intentions show how motherhood is repeatedly invoked within fatherhood-centered discussions as a background reference through which paternal involvement, maternal labor, and reproductive responsibility are evaluated.

**Conclusion:**

This study suggests that the visibility of fatherhood on social media is shaped by platform logics, algorithmic amplification, and user interaction. It introduces the concept of “endogenous symbolic annihilation” to explain how everyday praise, comparison, silence, and compliance in user interaction may reproduce a gendered hierarchy in which paternal involvement becomes exceptional, while maternal labor and reproductive experiences remain less explicitly valued. This extends symbolic annihilation from media representation to participatory digital interaction. It also contributes to debates on fathers as coparents by showing that the celebration of paternal involvement does not necessarily produce coparenting equity. In the Chinese context, it may reproduce a gendered hierarchy in which fatherhood is valued as exceptional, while motherhood remains naturalized as obligation.

## Introduction

On Chinese social media, short videos of fathers caring for their children often receive considerable attention. In the comment sections, users tend to praise fathers’ involvement in childcare, while mothers’ labor is often less explicitly recognized. These responses suggest that social media has become an important site where fatherhood is made visible. They also indicate that shared understandings of gendered childcare labor are being reshaped through platform interaction. The gendered division of labor in traditional Confucian culture, understood here as family norms emphasizing paternal authority and women’s domestic caregiving responsibilities, has deep historical roots in China. Within this framework, the idea of “men working outside the home and women managing the household” has remained a strong cultural norm in China (Yang, 2023). At the same time, China is undergoing both demographic transition and changes in gender norms. The childbearing-age population fell from 17.86 million in 2016 to 7.92 million in 2025 (National Bureau of Statistics of China, 2026). As a result, questions about child-rearing and the gendered division of labor have moved to the center of public debate. Meanwhile, the spread of feminist discourse in China (Chang, 2025) has encouraged greater public reflection on traditional gender divisions in childcare and the maternal burden behind them. Platforms such as Xiaohongshu have also become important spaces for the contestation of gender discourse (Zhao et al., 2025).

Social media does not present fathers’ involvement in childcare in a neutral way. Instead, platform mechanisms, audience interaction, and commercial logics work together to make fatherhood more visible and present it as a parenting practice that deserves praise (Scheibling, 2019; Xu et al., 2025; Liu et al., 2026). Research also suggests that the visibility of fatherhood does not simply appear. It is often produced through contrast with motherhood. Mothers are usually expected to take primary responsibility for childcare, while fathers’ relatively exceptional participation is more likely to be noticed and praised (Scheibling and Milkie, 2023; Shen and Jiao, 2024). However, most existing research still focuses on platform content and macro-level discursive frameworks. Few studies examine micro-level interactive spaces such as comment sections. Even fewer studies explore how ordinary users, through everyday acts of praise, comparison, persuasion, and affirmation, may increase the value of fatherhood, make maternal responsibility seem natural, and reproduce gendered ideologies of childcare. Rather than comparing fatherhood- and motherhood-centered posts, this study treats fatherhood performance as an interactional context in which motherhood is also invoked, compared, and differentially valued.

In this study, “discourse” refers not only to individual comments, but also to recurring patterns of language, evaluation, and social meaning through which users make sense of fatherhood, motherhood, and childcare responsibility. Building on these concerns, this study draws on public comments from Xiaohongshu, a Chinese social media platform, and combines thematic analysis, critical discourse analysis (CDA), and symbolic annihilation theory to explore how user interaction in fatherhood-centered comment threads may contribute to the construction of gendered meanings around fatherhood, motherhood, and childcare responsibility (Fairclough, 1995; Tuchman, 2000). CDA is used to examine the implicit assumptions and social meanings embedded in the comments. Symbolic annihilation theory provides a framework for interpreting whether and how motherhood is backgrounded, devalued, or naturalized through the increased visibility of fatherhood and everyday user participation.

This study addresses two research questions. First, what core discourses of “ideal fatherhood” appear in the comment sections of short videos about fatherhood? Second, to what extent do these public discourses reshape the visibility and value hierarchy of fatherhood and motherhood in childcare through the continued celebration of fatherhood?

## Literature review

### Gender roles and the construction of motherhood in Chinese childcare culture

Shaped by traditional Confucian patriarchy, Chinese childcare culture has long followed the gendered division of labor expressed in the saying “men manage external affairs while women manage the home.” Within this framework, mothers take primary responsibility for childcare, while fathers’ involvement remains limited (Yang, 2023). Traditional Confucian culture defines motherhood through the ideal of the “virtuous wife and good mother” and places women in the private domestic sphere. Through ideological discourses of filial piety and maternal instinct, childbirth and childcare are made to appear as women’s natural duties (Xie, 2019).

With China’s broader social transformation, ideas about gender roles have begun to change. In China, higher levels of education and employment, together with the growth of digital media, have weakened the traditional logic of gender roles in the family to some extent (Zhang and Liu, 2022). However, women remain the main caregivers in everyday parenting practice. Shen and Jiao (2024) describe fathers’ long-term absence from early child-rearing as a form of single-mother-style parenting. Their analysis shows a tension in Chinese parenting culture on social media platforms. On the one hand, it reinforces the naturalization of maternal labor under traditional gender roles. On the other hand, it also encourages some public reflection on the division of labor within the family.

Feminist consciousness among Chinese women has emerged on digital platforms. However, its impact on the division of childcare labor remains limited. State censorship, traditional culture, and commercial interests continue to restrict its influence. In this context, such discourse may still be drawn into the state’s family-governance agenda centered on marriage and childbirth (Zhang and Tang, 2026). The neglect of maternal labor is not limited to China. It can also be seen in broader global contexts. For example, although Norway has adopted a “dual-earner” model and paternity leave, related research and policy still focus mainly on the “second shift.” This leaves mothers to take on the “third shift” and other childcare responsibilities in less visible ways (Egeland et al., 2025). Similarly, a study of women’s unpaid labor in India found that women spend 75 min more per day on caregiving than men. This leads to much lower levels of life satisfaction, well-being, and health (Sinha et al., 2024). Taken together, these global comparisons show that the invisibility of maternal labor is a structural issue across different societies. However, they also highlight China’s particular complexity. Shaped by compressed modernity (Ji et al., 2017), Chinese mothers face both traditional patriarchal expectations and the neoliberal pressures of intensive mothering (Wang et al., 2024). This dual burden makes Chinese digital platforms a highly contested site for negotiating maternal labor.

Recent studies on Chinese digital motherhood further show that motherhood-centered visibility does not necessarily translate into recognition of maternal labor. On Douyin, stay-at-home mothers make domestic, affective, and entrepreneurial labor visible through short videos, yet this visibility remains entangled with platform commercialization and gendered care work (He et al., 2022). On Xiaohongshu, postpartum recovery discussions discipline mothers through ideals of bodily recovery, childcare responsibility, and self-surveillance rather than simply praising maternal labor (Liu and Wang, 2023). Research on “widow-style parenting” further shows that even discourses criticizing paternal absence may reinforce gender essentialism, blame mothers, and increase their burden, while reader comments simultaneously open a space for structural critique and resistance (Shen and Jiao, 2024). Similarly, mom creators’ narratives of reproductive injury and postpartum pain may become publicly visible, but such visibility is shaped by platform governance, affective labor, and creator-economy logics (Sun, 2026). These studies suggest that the central issue is not whether motherhood appears as a topic, but how maternal labor is valued within platform interaction.

On social media platforms shaped by neoliberal logics of individual responsibility and self-improvement, the double burden of motherhood is often presented as a personal project rather than a structural problem. This framing hides less visible forms of maternal labor, including emotional and cognitive labor (Alkan et al., 2025). Modern social media platforms have therefore become important sites where gendered parenting labor is reshaped. They trigger social comparison and reshape users’ psychological evaluations of parental roles. Scholars of postfeminist media culture have similarly shown that women’s own discursive practices can reproduce gender inequalities. These practices range from the normalization of femininity through individual choice (Gill, 2007), to the neutralization of feminist critique within mainstream media (McRobbie, 2009), and to the reinforcement of recognition hierarchies through women’s affective labor on digital platforms (Duffy, 2017). These postfeminist critiques clarify the mechanism through which women may become complicit in their own marginalization. By actively celebrating performances of fatherhood through a rhetoric of choice and empowerment, female users may inadvertently neutralize structural critiques of unequal caregiving. In this way, they become unwitting agents in the symbolic annihilation of maternal labor.

### Social media, fatherhood performance, and gender discourse

Platformization, visualization, and algorithmic recommendation now strongly shape the production, circulation, and negotiation of gender discourse (Mary et al., 2024; Chang, 2025). In this context, social media has increasingly become a discursive space where fatherhood is constructed. It is not only a visible space where fathers share parenting experiences and negotiate parental identities. It is also a normative arena where “engaged fatherhood” is displayed, promoted, and normalized (Scheibling, 2019). On the Chinese social media platform Xiaohongshu, this process is intensified by its specific platform affordances. Algorithmic visibility on Xiaohongshu relies on a “traffic pool” logic that strongly rewards high-engagement interactions (Zhao, 2025). It is also shaped by a distinct visual culture that favors stylized spectacles over mundane realities (Yu, 2026). Because maternal labor is heavily normalized, it often lacks the novelty needed to trigger algorithmic amplification. Fatherhood is therefore promoted as a “new role” in parenting practice and framed as evidence of men’s personal growth and competent masculinity, while also being packaged as a hybrid performance that combines companionship, education, and commercial value (Liu et al., 2026; Xu et al., 2025). As a result, the platform’s recommendation system amplifies fatherhood as a marketable spectacle while filtering out the less visible realities of maternal caregiving.

At the same time, fatherhood performance on these platforms is regularly defined through contrast with motherhood. These discourses usually position mothers as primary caregivers and fathers as mainly responsible for providing for the family (Scheibling and Milkie, 2023). This view is close to the traditional Chinese gendered division of childcare labor, expressed in the idea that “men work outside the home while women manage the household.” Research also suggests that the participatory fatherhood displayed on platforms responds to feminist critiques such as “widow-style parenting” (Shen and Jiao, 2024). However, this response may also strengthen the stereotype that motherhood is natural and inborn. Therefore, fatherhood performance on social media has not necessarily created a more equal gender discourse. Instead, it makes fatherhood appear more valuable and further presents motherhood as a woman’s natural duty.

Existing research has recognized that social media algorithms make fathers more visible. However, it has not fully explained how this visibility is co-constructed and reinforced by users in everyday interaction. In other words, while platform affordances provide the structural basis, how do women, as active discursive participants, negotiate these platform narratives? Why is fatherhood continuously praised as a valuable practice in user comments, while motherhood remains treated as a natural duty that requires no special recognition? This study examines these micro-discursive processes to reveal the hidden mechanism of symbolic annihilation.

### The theory of symbolic annihilation: from traditional media to social media

The concept of symbolic annihilation originates from Gerbner and Gross’s work on television representation. At its core, media representation is not a passive record of reality. It is a symbolic act that shapes how people understand social life. Certain groups may lose social legitimacy when they are excluded, misrepresented, or devalued through symbolic forms (Gerbner and Gross, 1976). Tuchman later applied this concept to women’s representation in the media and identified three dimensions: absence, trivialization, and condemnation (Tuchman, 2000). In this framework, absence refers to the exclusion or limited visibility of women, trivialization refers to representations that treat women’s roles as less socially valuable, and condemnation refers to portrayals that morally devalue women or mark them as deviant. Together, these three dimensions show how traditional mass media make women less visible, devalue their social roles, and lower their moral status. Over time, these processes weaken women’s social agency.

Early research examined the politics of access in media narratives, where the public erasure of women becomes visible. This erasure appears not only in individual images, but also in women’s difficulty in being taken seriously within public discourse (Bybee, 1990). Later studies extended this perspective to audience-specific analyses of gender identity and media environments. They also argue that motherhood is not only represented, but also compressed and marginalized in popular cultural narratives (Åström, 2015).

With the rise of social media, the explanatory scope of this theory has expanded further. Studies show that platforms not only exclude specific experiences from algorithmic narratives through functional presets and design logics, but also reduce the visibility of female subjects. Even when women are present, their visibility remains constrained by technology and discourse (Andalibi, 2021). Research on the visibility of female athletes in public discourse also suggests that symbolic annihilation in the social media era is shifting from clear absence to a more hidden form of marginalized presence. In other words, women may become visible on these platforms, but they still lack control over their position as subjects of discourse (Boling et al., 2024).

As noted above, at the intersection of China’s Confucian gender order and social media technologies, motherhood is naturalized as a default and invisible duty. Fatherhood, by contrast, is shaped into a rare performance that is both praiseworthy and marketable. This study therefore takes fatherhood performance as its starting point. It examines how platforms rebuild the hierarchy of visibility in parental labor through representations of fathers’ childcare practices. It also explores how platform discourse may further contribute to a form of symbolic marginalization, present but voiceless. Unlike existing research that focuses on the internalization of individual values and the reproduction of norms (Bartky, 2015; Ridgeway, 2011), this study emphasizes the role of collective discursive interaction on social media. It aims to explore the extent to which women, as a discursive public, take part in the marginalization of maternal experience.

## Materials and methods

Xiaohongshu is one of China’s most influential platforms for lifestyle sharing and the circulation of idealized ways of living. It has 339 million monthly active users (Xu et al., 2025). Its comment sections are also important sites for observing social attitudes and forms of collective consciousness in everyday interaction. Based on public comments on fatherhood performance on Xiaohongshu, this study examines how such discourse shapes the meaning of fatherhood and may help reproduce gendered meanings of childcare labor.

Data collection focused on the core keyword “daddy raising kids.” Because this keyword likely retrieved posts featuring relatively involved fathers, the analysis focuses on evaluations of visible fatherhood performance rather than the overall prevalence of paternal involvement on Xiaohongshu. This sampling strategy was not designed as a matched comparison between fatherhood- and motherhood-centered content. Instead, it was used to examine how users evaluate paternal caregiving and how motherhood is invoked, backgrounded, or differentially valued within fatherhood-centered discussions. Therefore, the analysis does not treat the mere topical focus on fathers as evidence of maternal invisibility. Following Campbell and Farrell’s influencer hierarchy model, posts were selected from accounts with 1,000 to 100,000 followers, including nano- and micro-influencers. This range was chosen because it offers higher perceived authenticity and greater community proximity than celebrity accounts. Only posts with at least 300 likes were included to ensure sufficient algorithmic visibility and organic reach (Campbell and Farrell, 2020). The data collection period ran from June 2024 to June 2025. This timeframe made it possible to identify stable discursive patterns rather than short-term fluctuations. This selection process yielded 53 qualifying posts, which generated an average of 56 comments each. The number of comments ranged from 12 to 185, with a median of 48. Initial scraping using a Python program yielded 2,975 comments. After comments containing only emojis or lacking substantive meaning were removed, 2,798 valid comments were retained as the primary units of analysis.

Since commenters’ actual gender identities cannot be directly verified in anonymous or pseudonymous online environments, this study adopts a contextual assessment approach. It triangulates explicit self-disclosures in the comment texts, such as “as a mother” and “my husband,” with publicly available account information, including nicknames, profile pictures, and gender markers in user bios (Kopf, 2026). Based on this assessment, approximately 78% of the comments (*n* = 2,182) were attributed to female discursive positions, 12% (*n* = 336) to male positions, and 10% (*n* = 280) remained ambiguous. Among the valid comments, 2.6% (*n* = 73) were classified solely on the basis of nicknames and/or profile pictures, and all gender-coded classifications were used only to describe discursive positioning rather than verified gender identity. The dominance of female-coded discourse supports the relevance of this dataset for examining women’s own communicative practices.

This study adopts Fairclough’s three-dimensional framework of critical discourse analysis to examine public comments on Xiaohongshu across three dimensions: text, discursive practice, and sociocultural practice. The textual dimension examines how discursive meaning is constructed through linguistic forms, such as lexical choice and grammatical structure. The discursive practice dimension focuses on the production and consumption of texts, especially how they circulate in specific contexts and how users interpret and respond to them. The sociocultural dimension places texts within broader social and political-economic contexts. It explores how micro-level discourses maintain, reconstruct, or challenge existing gender structures and power relations (Fairclough, 1995). For example, the analysis examined textual markers such as “cosmetic surgery,” “only natural,” and “duty,” interactional tensions between “conscious resistance” and “compliant discourse,” and broader links to Confucian gender norms, platform commercial logics, and low-fertility pressures. Table 1 summarizes this analytical process.

**Table 1:** Summary of the three levels of critical discourse analysis.

CDA dimension	Analytical focus	Analytical indicators
Text	Use of complimentary vocabulary, covert rhetoric of maternal devaluation, and syntactic structures of conditional fertility intentions.	Identifying evaluative vocabulary, naturalizing expressions, metaphoric descriptions, and conditional sentence structures used in comments.
Discursive practice	Discursive tension in comment sections, contrastive framing of fatherhood and motherhood, and the micro-negotiation of female subjectivity.	Examining how comment threads contrast fatherhood and motherhood, how users respond to fatherhood-centered content, and how resistant and compliant comments negotiate the meanings of childcare labor and female subjectivity.
Sociocultural practice	Traditional Confucian gender ideology, platform traffic and monetization logics, and gender politics amid demographic transition.	Interpreting how the identified discursive patterns relate to Confucian gender norms, platform commercial logics, low-fertility pressures, and the changing forms of symbolic annihilation in social media parenting discourse.

This study combines critical discourse analysis with the theory of symbolic annihilation to strengthen its analytical framework. Critical discourse analysis focuses on how discourse conceals power relations and serves ideological functions. From a cultural and sociological perspective, it also examines how texts reinforce or transform social structures. The theory of symbolic annihilation helps explain how the celebration of fathers’ childcare practices can make mothers’ labor appear less visible and less valued (Gerbner and Gross, 1976; Tuchman, 2000). Used together, these two approaches make it possible to examine how public comments construct gendered meanings of childcare. They also help examine how such comments may reproduce social beliefs about fatherhood, motherhood, and parental responsibility.

The analysis integrated thematic analysis and critical discourse analysis within an interpretive framework informed throughout by symbolic annihilation theory. Symbolic annihilation theory guided the interpretation of which recurring patterns were theoretically significant, rather than being applied as a separate coding step after the themes were finalized. Thematic analysis was first used to identify recurring interpretive patterns across the comments (Braun and Clarke, 2021), producing a preliminary thematic map. These patterns were then examined in greater depth through Fairclough’s three-dimensional CDA framework, focusing on textual features, discursive practices, and broader sociocultural meanings (Fairclough, 1995), to assess how the identified discourses positioned fatherhood, motherhood, and childcare responsibility.

To establish analytical consistency, two coders independently coded a randomly selected 10% sub-sample (279 comments). The 10% sub-sample was selected as a calibration set because it was large enough to include comments from all 53 sampled posts while remaining manageable for detailed interpretive comparison. Initial codes and thematic boundaries were then compared and refined through iterative discussion. Here, “thematic boundaries” refers to the criteria used to decide whether similar comments belonged within the same theme or should be separated into different themes. For example, comments praising fathers as unusually competent caregivers were distinguished from comments linking fatherhood performance to commercial visibility, even though both involved positive evaluations of fathers. Following Braun and Clarke’s (2021) approach to thematic analysis, inter-coder agreement was reached through collaborative negotiation rather than statistical metrics such as Cohen’s Kappa, because thematic analysis treats coding as an interpretive process rather than a purely classificatory one.

After the coding framework was finalized, it was applied to the full sample. One coder coded the remaining comments, while the second coder reviewed theme allocation, ambiguous cases, and examples selected for presentation. Remaining disagreements were resolved through discussion until consensus was reached. Codes were merged when they referred to the same underlying interpretive pattern and dropped when they appeared only incidentally, lacked relevance to the research questions, or could not be meaningfully linked to the study’s theoretical framework. Themes were identified not by frequency alone, but by their recurrence across posts, interpretive depth, and theoretical relevance to symbolic annihilation. This process yielded four main themes. Thematic analysis identified the broad discursive patterns, while the subsequent CDA stage deepened their interpretation by clarifying how each pattern operated linguistically, interactionally, and socioculturally. In some cases, CDA also refined the boundaries between themes as their discursive mechanisms became clearer.

This study does not treat Xiaohongshu comments as transparent expressions of users’ individual beliefs or fertility intentions. Rather, comments are understood as situated discursive performances shaped by Xiaohongshu’s platform-specific communicative norms, including hyperbole, humor, irony, and formulaic expressions. This approach is consistent with studies showing that Xiaohongshu discourse is shaped by platform infrastructures, interactional conventions, visibility norms, and affective performance (Liu and Wang, 2023; Sun, 2026). Drawing on Austin’s (1962) speech act theory, this study distinguishes the literal propositional content of such comments from their pragmatic and performative force in interaction. Comments such as “With a husband like that, I’d be happy to have three kids” are therefore analyzed not as literal reproductive intentions, but as evaluative and formulaic ways of praising idealized male caregiving. Their analytical significance lies in their cumulative discursive function across comment threads, rather than in the sincerity of any individual commenter.

To protect commenter privacy (Xiaohongshu, 2025), all user IDs have been anonymized in the presentation of results, and all quoted comments have been paraphrased in translation to minimize traceability while preserving discursive intent. This practice follows Markham’s (2012) principle of ethical fabrication and is informed by Sun (2026), whose approach demonstrates that paraphrasing can effectively reduce the risk of reidentification in China’s highly searchable digital environment. Illustrative quotes were purposively selected based on their representativeness of the core discursive logic within each theme. They were also selected for their capacity to articulate the nuanced social attitudes found across the 2,798 comments.

## Results

Overall, the analysis identifies a recurring pattern in which evaluative terms and conditional expressions mark fatherhood as exceptional while maternal labor is often naturalized. At the discursive and sociocultural levels, this pattern is further examined through resistant and compliant interactions and in relation to Confucian gender norms, platform commercial logics, and fertility pressures in China.

Table 2 reports the distribution of comment counts across themes and gender-coded discursive positions.

**Table 2:** Comment counts by theme and gender-coded discursive position.

**Theme no.**	**Theme / subtheme**	**Total comments**	**Female-coded**	**Male-coded**	**Ambiguous**
Theme 1	The Construction of the “Ideal Father” in Discourses of Praising Fatherhood	1,356	1,048	184	124
Theme 2	The Commercialization of Fatherhood and the Devaluation of Motherhood	526	421	56	49
Theme 3a	Conscious Resistance	186	171	4	11
Theme 3b	Compliant Discourse	613	517	47	49
Theme 4	Conditional Fertility Intentions	704	650	20	34

Theme counts are not mutually exclusive because some comments performed more than one discursive function.

### The construction of the “ideal father” in discourses of praising fatherhood

Online comments are saturated with praise for fathers who care for their children. This praise focuses on physical appearance, masculine responsibility, and perceived qualifications for fatherhood. On the one hand, it reflects users’ expectations of the ideal father. On the other hand, it draws attention to how mothers’ roles and maternal labor are less explicitly recognized within these fatherhood-centered discussions. Because the sample was intentionally centered on fatherhood-related posts, the analysis does not treat the limited explicit discussion of motherhood as evidence of absence in itself. Instead, it focuses on how maternal labor is positioned when it is invoked within fatherhood-centered praise.

First, users treat men’s involvement in childcare not only as worthy of praise, but also as a source of masculinity and charm. In this sense, male childcare becomes a spectacle. In Chinese parenting culture, women have traditionally played the main role in child-rearing. Although ideas of gender equality have become more influential in Chinese society, men’s childcare is still often seen in everyday parenting culture as an “exception” rather than the norm. For example, one commenter responded to a fatherhood video by asking, “Am I hallucinating? Are there really men like this?” (@Xiao, 23 May 2025). When users see videos of men caring for children, they consistently offer praise. Typical comments include “It’s so cool to see a man handling childcare so efficiently” (@Cui, 17 August 2024) and “It turns out that being a good father is the best ‘cosmetic surgery’ for a man” (@momo, 15 May 2025). Here, the language of cosmetic surgery, which is usually associated with female consumer culture, is used to describe men’s parenting behavior. On the one hand, it frames male parenting as an active choice that increases personal value. At the same time, maternal labor is not brought into the same logic of praise in these comments. These comments clearly connect male childcare with masculine charm and reinforce the gendered division of childcare labor in Chinese culture. As praise for fathers’ caregiving continues, maternal care and its value receive less explicit attention within the thread.

Second, the detailed caregiving practices shown by male bloggers further lead users to frame male parenting through a logic of professionalism, responsibility, and moral worth. The division of parenting responsibilities has long been debated on Chinese social media, and men’s absence from parenting has even been described as “widow-style parenting” (Shen and Jiao, 2024). When detailed and wide-ranging caregiving by men is displayed, users often use terms such as “professional,” “responsible,” and “great dad” to express approval. For example, one user commented, “This dad does a better job than even a top-tier postpartum care worker” (@Lynn, 8 December 2024). Another wrote, “No need for a postpartum care worker or help from the elderly—raising the child entirely on his own—that’s what a truly good dad looks like” (@Meng, 1 November 2024). Another user stated, “He really is a responsible father; the fact that Chinese men are beginning to recognize the importance of parenting is a sign of social progress” (@Chen, 3 May 2025). These comments show approval of changing gender roles in parenting.

Third, the ideal father is presented as a figure who can reduce women’s fear of childbirth and even change their willingness to have children. Qiao et al. (2024) show that women’s lower expectations of motherhood and reluctance to have children increase as feminist identity becomes stronger. In the comments, however, women often state that they would be more willing to have children if their partners were actively involved in parenting. One user wrote, “This is the standard dad; if he can be like this, then we can talk about having kids, and I will not be afraid of childbirth anymore” (@Nuo, 19 July 2024). In this comment, good fatherhood becomes a condition for reducing fear of childbirth. A similar view appears in the comment, “If there were more husbands like this, I bet our country’s birth rate would skyrocket” (@Feng, 9 February 2025). Here, the ideal father is imagined as a possible solution to the problem of low fertility. This logic of praise may shift attention away from women’s labor in childbirth and childcare and from the structural difficulties women face in reproduction.

Overall, praise for men’s childcare, approval of their sense of responsibility, and expressions of personal fertility intention together reveal the complex ways in which users, especially women, understand men’s involvement in childcare. These discourses reflect the gender inequalities embedded in China’s childcare practices. They suggest that men’s public display of parenting can make maternal labor less explicit as a topic of recognition, while some female-coded comments give limited attention to the value of women’s own caregiving work.

### The commercialization of fatherhood and the devaluation of motherhood

Fathers’ parenting roles are often framed as performative and commercially valuable. In this process, maternal labor tends to be positioned as necessary work with less performative or monetizable appeal. For example, comments such as “I love watching dads care for their children with such fluid, effortless movements; it leaves me feeling refreshed and at ease” (@Wang, 9 November 2024) reflect this logic. Another user wrote, “Even though it’s just a video, he must have practiced a million times late at night. It’s truly amazing” (@kcl, 17 February 2025). The words “effortless” and “fluid” present fathers’ childcare as both technical and aesthetic. They also emphasize the difficulty behind this labor, making it appear as skilled work that deserves praise. By contrast, maternal care is less often framed through the same performative or monetizable logic in this dataset. Some users understood this performance as a sign of gender equality. One commented, “Whether this is a performance or not, at least the dad is doing something, which is good for gender equality” (@Lily, 25 January 2021). By emphasizing the value of good fatherhood, users differentiate fatherhood from motherhood. This distinction may contribute to a hierarchy of recognition in which motherhood appears to carry less performative value.

Compared with motherhood, “daddy bloggers” are seen as having greater performative value. This leads users to view their caregiving content as easier to monetize on social media platforms (Yu, 2026). For example, one commenter stated, “Dad bloggers have it so good—they can just have a kid and make money by filming videos, unlike me” (@Yi, 11 December 2024). Although the commenter’s parental status cannot be verified, the comment contrasts the visibility and monetizability of dad-blogger content with less visible forms of caregiving labor. This recurring self-deprecating discourse suggests that some female-coded comments may reproduce an unequal standard of labor evaluation, in which fatherhood performance appears more visible and rewardable than routine caregiving labor. Another commenter stated, “It’s no longer ‘men work outside the home while women manage the household’—it’s ‘men work outside the home and men manage the household’ now. So I do not have to bother anymore; I’ll just give up and go with the flow” (@Wen, 22 September 2024). This comment further recasts parenting labor as a role that men can take on and benefit from. At the same time, motherhood is positioned more as background labor than as a similarly visible or monetizable practice.

Within China’s traditional gender ideology, men are encouraged to pursue career success, while women are expected to become virtuous wives and good mothers (Chen, 2024). Against this background, some female-coded comments frame the monetization of childcare as comparable to men gaining the rewards of career success. What is striking, however, is that men are now seen as entering a domain traditionally assigned to women in the gendered division of labor. Traditional gender roles are therefore being reshaped through childcare discourse. This process highlights the wider recognition of men’s caregiving labor while suggesting that maternal labor may be granted less explicit value in these discussions.

Overall, public discourse strongly values the performative and commercial dimensions of fathers’ childcare. In doing so, it indirectly reveals the lower economic value assigned to mothers’ childcare labor. As one commenter stated, “Fathers raising kids can make way more money than mothers. It is only natural for mothers to take care of their children, but fathers raising kids is truly a unique sight” (@Yan, 5 February 2025). Phrases such as “only natural” and “a unique sight” illustrate how maternal labor may be naturalized while fatherhood is marked as exceptional. This gap does not appear through an explicit denial of motherhood. Instead, it emerges through the continued commercialization of fatherhood, which fixes maternal labor as a natural duty that does not need to be seen and cannot be monetized. This is a specific expression of the devaluation dimension of symbolic annihilation in social media parenting discourse. Maternal labor is not directly denied. Rather, it is granted less discursive visibility and economic value within continued attention to the spectacle of fatherhood.

### “Conscious resistance” and “compliant discourse” within discursive tensions

In the Chinese cultural context, motherhood is closely linked to child-rearing. As a result, maternal labor is often treated as a duty rather than as work that deserves recognition. When fathers’ involvement appears on social media as a performance, this structure is not simply challenged. Instead, it creates a more complex discursive tension, prompting women to reflect critically on their own position. Such critical reflection does appear in the dataset, but it remains peripheral: resistant comments represent a small minority within threads overwhelmingly shaped by praise and compliance. They are included here to illuminate the contested nature of the discursive space and, in so doing, to underscore the dominance of the surrounding symbolic annihilation.

This “conscious resistance” typically emerges when the idealized spectacle of fatherhood serves as a mirror, prompting women to recount their own lives and recognize how maternal labor has long been ignored. One user wrote, “I have to work and take care of the kids; only I know how exhausting those sleepless days and nights have been” (@Nian, 10 November 2024). Resistance is often sparked by the perceived privilege of male “influencer” parenting. In one exchange, a user challenged the authenticity of the performance by asking, “Does the dad not work?” (@Rev., 11 March 2025), attempting to puncture the myth of the “ideal father.” However, this resistance is often immediately neutralized by the platform’s commercial logic. “Recording videos is his job,” a compliant reply countered (@Can, 14 March 2025). This interaction shows how critical questioning of the gendered division of labor can be silenced by reframing performance as a legitimate professional role. This critical stance often triggers a broader re-evaluation of gendered responsibilities. For instance, one commenter questioned the assumption that childcare should fall primarily on mothers by stating, “Shouldn’t the dad help with the kid? They both have rights to the child” (@Yu, 24 April 2025). Within these discursive spaces, discussions of childbearing are increasingly intertwined with feminist ideas. One user wrote, “Do not ignore or take for granted the work of a mother; it should be praised just as much as the work of a father in raising a child” (@Qin, 27 May 2025). Women are becoming more aware of how Chinese parenting norms shape their lives after they become mothers, and they are increasingly willing to speak about this experience.

On the other hand, this resistance often fails to disrupt the dominant narrative because it is immediately countered by compliant discourse within the same comment threads. The failure of resistance is often rooted in a low-bar logic that treats any male participation as a moral triumph. In the Chinese context, fatherhood performance can make gendered labor, especially women’s childcare labor, less explicit within the thread. At the same time, some female-coded comments become part of this discursive process. They often use duty as a moral shield to dismiss critical voices. For example, one commenter wrote, “I think taking care of the child is a mother’s responsibility. Of course, if the father looks after the child occasionally, that’s fine; I’d be happy if he could help share the burden” (@He, 3 December 2024). This compliance is reinforced by the perceived scarcity of responsible fathers in China. As one user noted, “Because so many fathers are irresponsible, doing the bare minimum is seen as excellent” (@Xiang, 5 February 2025). This low-bar logic acts as a discursive containment mechanism. When critics point to the structural invisibility of mothers, they are often countered by the view that a “present” father is a rare gift that should not be scrutinized. Another commenter expressed a similar view: “Fathers should still focus on earning money to support the family, while I’ll fulfill my duty as a full-time mother” (@Xian, 6 February 2025). Here, the repeated use of “duty” turns the gendered division of childcare into a moral obligation. It makes this division appear natural rather than socially constructed. Some commenters also argued, “For a father to both care for a child and work is likely unfeasible under current social pressures in China” (@Yin, 28 September 2024). One commenter even stated, “When I was a child, my mother and grandmother raised me while my father worked outside the home. Rather than going out to work, I’d prefer to stay home and care for my child” (@Ran, 23 November 2024). These statements suggest the continued resonance of traditional gender roles in some female-coded comments. They also connect the existing labor division to practical constraints and historical conventions. Within China’s traditional gender order, women are positioned as primary caregivers. They not only locate themselves within this arrangement, but also link it to broader ideas of social development and child-rearing custom. In this way, compliant discourse acts as a containment mechanism. It helps sustain a father-centered evaluative frame in which the value of maternal labor remains less explicit. The asymmetry between the volume of compliant discourse and the scarcity of resistant voices supports the interpretation of endogenous symbolic annihilation: resistance exists, but remains structurally peripheral.

Fatherhood performance on Xiaohongshu further complicates discourse on gender roles in contemporary China. In response to both women’s “conscious resistance” and the “compliant discourse” around traditional gender norms, comment sections reveal a hidden logic of symbolic annihilation in the social media context. This tension is not fully resolved in the comment sections. Rather, conscious resistance and compliant discourse coexist, producing a contested discursive space in which women’s agency and the marginalization of maternal labor appear simultaneously. The devaluation of maternal labor is therefore not simply imposed from outside, nor is it reducible to women’s individual internalization. It is reproduced through interactional practices in which women both challenge unequal caregiving arrangements and, at times, rely on father-centered terms to make those challenges intelligible. As a result, its reproductive effects become deeper and more lasting.

### The suspension of women’s reproductive experience in conditional fertility intentions

China is now facing both low-fertility pressure and the spread of feminist discourse. As fatherhood performance enters this discursive space, childbearing is repeatedly brought back into online comment sections. However, these discourses do not place women’s bodily experiences and reproductive difficulties at the center of discussion. Instead, they focus on the evaluation and imagination of men’s parenting behavior. As a result, the embodied costs of childbearing receive less explicit attention in these comment threads. Unlike the more direct absence of maternal labor shown in the first three findings, this mechanism is more hidden. Women actively participate as discursive subjects, and their comments often express conditional reproductive agency. However, this agency is frequently articulated through evaluations of men’s parenting behavior, which may shift attention away from women’s embodied reproductive labor.

Conditional fertility intention functions not as an isolated sentiment, but as a pervasive discursive repertoire across comment sections. @You (25 July 2024) stated, “With a husband like that, I’d be happy to have three kids.” @Rui (22 March 2025) similarly wrote, “With a husband like that, who would not want to keep having kids until menopause?” This pattern is further reinforced by a collective rhetoric that links male performance to macro-social indicators such as national birth rates. For instance, commenters frequently asserted, “With a dad like this, who would dare not to marry and have kids?” (@Jia, 2 March 2025), or suggested that “Teammates like this will only increase the birth rate” (@Cue, 23 August 2024). Some even jokingly called for state intervention: “If the state gave out husbands like this, would not the marriage rate go up?” (@Meng, 16 October 2024). These comments follow a highly routinized logic. Women’s reproductive decisions are framed as increasingly contingent on the husband’s performance. In this model, conditional expressions such as “I’d be happy to” can be read as a form of agency, because they set conditions for reproduction and demand more responsible fatherhood. At the same time, this agency is framed through the evaluation of men’s conduct, making paternal performance the central reference point for imagining childbearing.

These conditional statements can be interpreted as a pragmatic rhetorical strategy. They allow women to advocate for shared parenting by using fertility intention as a form of discursive power (Li and Zhou, 2025). This should be understood as a form of ambivalent empowerment rather than a simple form of self-erasure. Women use fertility intention as discursive leverage to demand shared parenting, but this leverage remains tied to a father-centered evaluative frame. By framing men’s performance as a primary condition for childbearing, this rhetoric calls for shared childcare while giving comparatively less explicit attention to the bodily trauma, postpartum risks, and biological costs specific to women (Hao et al., 2026). In these discursive exchanges, the physical experiences of pregnancy, childbirth, and breastfeeding are rendered secondary to the moral evaluation of the father. These experiences constitute reproductive labor that men cannot fully replicate or replace. As a result, women’s embodied reproductive experience is often placed in a secondary position within these discussions.

Yet, beyond the dominant discourse of conditional childbearing, a small number of comments still directly mention the physical burden of pregnancy. For example, one commenter wrote, “You have to carry the baby yourself for those nine months; in the third trimester, I could not breathe or sleep for nights on end” (@Sui, 25 May 2025). Another stated, “I’m still in the postpartum recovery period, and my bones already ache—yet I’m still being pressured to breastfeed” (@Pao, 26 July 2024). These detailed accounts of childbirth and caregiving show that women remain clearly aware of the physical burden of reproduction. These visceral narratives of bodily trauma stand in sharp contrast to the romanticized and hypothetical tone of conditional fertility statements. However, within the digital spectacle of the “ideal father,” these raw and less shareable realities of maternal suffering are implicitly treated as disruptive or out of place. Such voices do not become widespread in the comment sections. Instead, they remain marginalized within the dominant discourse of conditional fertility intention. Alongside the overwhelming celebration of male participation, they continue to occupy a secondary position in the broader conversation.

Overall, in the comment sections of short videos showing fathers caring for children, women have not stopped talking about marriage and childbearing. However, these voices do not always place women’s own experiences at the center of reproduction. When discussing childbearing, these comments often give less explicit attention to women’s own experiences and the physical burden of childbirth. Instead, they treat their evaluation of men as a key condition for their willingness to have children. This does not negate women’s reproductive agency. Rather, it shows how such agency is articulated within a father-centered evaluative frame, which may partially displace women’s embodied reproductive experience from the center of discussion.

## Discussion and conclusion

Returning to the two research questions, the findings first show how users construct an “ideal father” through praise, professionalized evaluation, and conditional fertility talk. They also suggest that these fatherhood-centered discourses may reshape the visibility and value hierarchy of fatherhood and motherhood by making paternal involvement exceptional while leaving maternal labor less explicitly recognized. The findings suggest that comments on short videos about fatherhood performance can contain forms of symbolic annihilation related to women’s labor, value, and reproductive agency. More specifically, the spectacularization of fatherhood may make motherhood less explicitly recognized within fatherhood-centered discussions. This relative lack of explicit recognition may create conditions under which maternal labor is more easily devalued or naturalized. This devaluation may then be reproduced through everyday discursive practices, including some female-coded comments, and may contribute to the partial displacement of women’s embodied reproductive experience. These findings suggest that symbolic annihilation in the social media context may work not only through traditional neglect (Tuchman, 2000), but also through overlapping patterns of differential recognition, naturalization, and interactional reproduction. Rather than a fixed sequence, these patterns point to several ways in which maternal labor and reproductive experience may become less explicitly recognized within fatherhood-centered discussions. Thus, the study does not make a frequency-based comparative claim about fatherhood- and motherhood-centered content; rather, it shows how maternal labor is positioned within fatherhood-centered interaction.

China’s traditional gender ideology is rooted in Confucian culture. It gives women primary responsibility for caregiving and places men in the role of family providers (Yang, 2023). When images of fathers caring for children appear repeatedly on social media, they come into conflict with traditional gender roles. They therefore become spectacularized. Under the commercial logics of social media platforms, and with the further amplification of technologies such as algorithms (Zhao, 2025), this spectacular content increases the visibility of fatherhood discourse. At the same time, it may make maternal labor less visible within the discursive field of childcare.

This study does not claim that motherhood is entirely absent from comment sections. Indeed, maternal labor is frequently invoked, but its invocation is constitutively different from recognition. Following Tuchman’s (2000) tripartite framework of absence, trivialization, and condemnation, the mechanism identified here most closely approximates trivialization, but with a participatory twist. Maternal labor appears as the unmarked background against which paternal performance is measured and celebrated. The distinction between irrelevance and annihilation is therefore not a matter of presence versus absence, but of discursive positioning. Motherhood is present as the assumed norm, fatherhood is present as the praiseworthy exception. It is this asymmetric framing, rather than mere silence alone, that this study interprets as a form of symbolic annihilation in participatory digital contexts.

Moreover, the “compliant discourse” found in female users’ comments reflects the long-term influence of traditional Chinese gender ideology on women’s own understandings of childcare. Women often praise fathers’ parenting while remaining silent about the value of their own labor. This reflects a postfeminist sensibility (Gill, 2007), in which female subjects actively participate in their own discursive discipline. By celebrating paternal involvement, women may both encourage more equal caregiving and reproduce a gendered recognition hierarchy in which paternal care is treated as exceptional while maternal care remains ordinary. This ambivalence resonates with McRobbie’s (2009) account of the undoing of feminism, where agency may coexist with the reproduction of gendered norms. At its core, this is a form of discursive discipline produced by the traditional gendered division of labor in the social media era. At the same time, the growth of feminist consciousness in China has allowed some women’s discourses to challenge traditional gender roles (Shen and Jiao, 2024). However, these voices do not fit the sensational commercial logic of Chinese social media platforms (Xu et al., 2025). Nor do they align with the dominant discourse reproduced by many female users under the influence of traditional culture. As a result, these resistant voices reveal the contested nature of the comment sections, but they do not fully displace the dominant evaluative framework in which fatherhood remains more readily praised and maternal labor remains comparatively less recognized. This coexistence of resistance and annihilation is theoretically consistent. Symbolic annihilation, as Tuchman (2000) conceived it, does not require the total elimination of counter-voices, but rather the systematic marginalization of an experience such that it cannot attain the same discursive weight as the dominant frame. The presence of resistant speech acts therefore does not necessarily undermine the symbolic annihilation interpretation; rather, their limited discursive weight helps explain why this interpretation remains plausible.

Against the backdrop of a continuously declining birth rate, childbearing remains a highly politically sensitive issue in China. Existing research has shown that factors such as high childcare costs, work-related stress (Wang et al., 2024), and the rise of feminism have shaped Chinese women’s awareness of the costs of childbearing (Qiao et al., 2024). However, in the context of short videos showing fatherhood, women’s conditional willingness to have children redirects this awareness toward the availability of responsible fatherhood. This dynamic echoes Duffy’s (2017) concept of aspirational labor, in which the pursuit of an idealized digital aesthetic requires the masking of messy reproductive realities. By asserting the right to judge men, women foreground the structural importance of paternal participation. Yet this assertion can also shift the center of discussion from women’s reproductive burdens to men’s parenting performance. This weakening of awareness reflects the operation of symbolic annihilation within the continuing cultural norm of “men working outside the home and women managing the household” in China (Ji et al., 2017).

In theoretical terms, this study makes two contributions. First, it extends the concept of symbolic annihilation beyond traditional mass-media representation analysis to the micro-level interaction practices of users on digital platforms. It shows that social media is not only a site where contemporary gender discourse is produced (Shen and Jiao, 2024; Chang, 2025), but also a space where gendered recognition hierarchies are reproduced through user interaction. More specifically, this study suggests that some female-coded discursive interactions may become one mechanism through which the relative invisibility and devaluation of motherhood are reproduced in less visible ways.

Second, by synthesizing postfeminist and digital labor perspectives with symbolic annihilation theory, this study shows how symbolic annihilation can be reproduced through participatory interaction, including by users who are themselves negotiating gender inequality. This departs from Gerbner and Gross’s (1976) original account of externally driven marginalization. It conceptualizes this interactional mechanism as endogenous symbolic annihilation. The concept of endogenous symbolic annihilation is analytically distinct from three related frameworks in terms of mechanism, site, and object of regulation. Bartky’s (2015) account of psychological oppression locates domination in the colonization of individual self-evaluation, whereby women internalize intimations of inferiority and the oppressor comes to reside within the self. By contrast, the process identified here does not primarily operate through women’s diminished sense of self-worth. Many female commenters show clear awareness of gendered inequity and even use fertility intention as explicit discursive leverage. The mechanism instead lies in collective symbolic accounting. Through the cumulative force of praise, selective silence, and conditional framing across comment threads, maternal labor appears to occupy a lower position within a shared public register of value. This process also differs from Ridgeway’s (2011) account of gender status beliefs and compliance practices. Ridgeway emphasizes widely shared cognitive frames that shape judgment and behavior in social relational contexts, often without conscious awareness. The practices examined here are more reflexive and ambivalent. Female commenters recognize and sometimes contest unequal care arrangements, yet they still reproduce a discursive hierarchy in which paternal caregiving appears exceptional while maternal labor remains ordinary and less visible. The concept also departs from Foucauldian self-surveillance. Rather than regulating individual conduct, self-monitoring, or self-perception, endogenous symbolic annihilation regulates the collective terms through which care work becomes symbolically recognizable in public discourse.

The term “endogenous” does not imply that external forces are absent. Platform algorithms, Confucian ideology, and commercial logics all shape the discursive field as distal structural conditions. What “endogenous” specifies is the proximate mechanism of annihilation. In Gerbner and Gross’s (1976) original account, annihilation was produced by media institutions through editorial selection and framing, processes external to women as social subjects. In the participatory context analyzed here, symbolic annihilation appears to be reproduced through users’ practices of praise, comparison, selective silence, and conditional framing. External structures create the conditions of possibility, but the specific comments are produced through users’ own interactional practices. To borrow Bourdieu’s (1990) formulation of structured improvisation, external structures establish the rules of the game, but the specific moves are made by the players themselves.

The comment sections under short videos of fatherhood performance do more than praise men for participating in childcare. They provide a space in which fatherhood and motherhood are differentially seen and valued. Maternal labor may become less visible, less valued, and more easily treated as a natural duty within these fatherhood-centered discussions. This study therefore combines critical discourse analysis with symbolic annihilation theory to examine how the treatment of motherhood on social media is shaped not only by platform logics and external discourse, but also by everyday communicative practices, including some female-coded comments. It introduces the concept of endogenous symbolic annihilation as a way to extend the theory from media representation to participatory digital interaction and collective discursive reproduction.

This study has some limitations related to data source and classification. Gender-coded classifications should be understood as contextual indicators of discursive positioning rather than confirmed gender identities. In addition, Xiaohongshu is mainly used by young and middle-aged urban women (Xu et al., 2025). Therefore, the findings should not be generalized directly to all Chinese social media users, rural populations, older users, or users on platforms with different demographic and algorithmic structures. Future research could examine other platforms, such as Douyin, to compare whether the mechanisms of symbolic annihilation are platform-specific or shared across platforms. Although this study does not include a matched comparison with motherhood-centered content, its focus is not on measuring praise rates across content categories, but on how motherhood is invoked and differentially valued within fatherhood-centered discussions. Future research could extend this work through a comparative design. The data were collected within a specific time frame, so this study cannot show how discourse changes over time. Future studies could use longitudinal data to examine how fatherhood discourse develops. Finally, although this study interprets some comments on short videos of fatherhood performance as reproducing forms of symbolic annihilation, discourses of “conscious resistance” still exist. Further research is needed to examine how these resistant discourses emerge and circulate. Such work would help explain how feminist consciousness seeks visibility within a discursive space shaped by symbolic annihilation.

## Data Availability

Although the data were collected from publicly accessible social media content, the anonymized dataset is available from the corresponding author upon reasonable request, subject to appropriate safeguards to protect user privacy.
